# Draft Genome Sequence of Bacillus cereus TN10, a Chromium-Resistant and -Reducing Strain Isolated from Tannery Effluent

**DOI:** 10.1128/MRA.00603-20

**Published:** 2020-07-02

**Authors:** Maqsud Hossain, Samiha Tasnim, Ashrafus Safa, Abdullah Bin Hossain Rayhan, Mohammad Tanvir Ibna Ashad Khan, Nayeema Bulbul, Abdus Sadique, Gias U. Ahsan, Jinath Sultana Jime

**Affiliations:** a Genome Research Institute, North South University, Dhaka, Bangladesh; b Department of Biochemistry and Microbiology, School of Health and Life Sciences, North South University, Dhaka, Bangladesh; c School of Life Sciences, Independent University, Bangladesh (IUB), Dhaka, Bangladesh; University of Rochester School of Medicine and Dentistry

## Abstract

Here, we present the draft genome sequence of Bacillus cereus strain TN10, which exhibited chromium resistance and chromium-reducing ability. The whole-genome sequence analysis of strain TN10 will help us to understand its genetic factors involved in the bioremediation of Cr^6+^.

## ANNOUNCEMENT

Hexavalent chromium (Cr^6+^) species are extremely mobile and highly toxic to humans and animals. Among others, tannery effluents are known as major sources of Cr^6+^ in the environment. To date, a number of aerobic and anaerobic microbes with Cr^6+^ bioremediation potential have been identified (1). Bacillus cereus strains, a group of Gram-positive, facultative anaerobes, are ubiquitous in nature, and strains isolated from wetlands, wastewater, and soils have exhibited prospective chromium remediation ability (2, 3). In this study, we report the draft genome sequence of Bacillus cereus strain TN10, which was obtained from tannery effluent. This strain is currently being explored as a microbial remediation candidate for Cr^6+^ removal from tannery effluents before they are discharged into the environment.

Bacillus cereus strain TN10 was isolated from tannery effluent according to a method described previously (4). Identification was confirmed by microscopic and biochemical analyses according to *Bergey’s Manual of Determinative Bacteriology* (5). TN10 was found to survive aerobically in 500 mg/liter potassium dichromate and was capable of reducing ∼87% of 18 μg/ml soluble Cr^+6^ in 24 h. A single colony of TN10 was grown overnight (18 h) with shaking (200 rpm) in 10 ml of Luria-Bertani broth (Becton, Dickinson) at 37°C. Five milliliters of this culture was centrifuged at 13,000 × *g* to harvest the cells, and the resulting cell pellet was resuspended in 1 ml of water. Genomic DNA was isolated using the blood and cell culture DNA minikit (Qiagen, Germantown, MD, USA), according to the manufacturer’s instruction. The genomic DNA was used to construct a whole-genome sequencing library using the Nextera XT DNA library preparation kit, according to the manufacturer’s instruction. The libraries were sequenced using the MiSeq platform (Illumina, San Diego, CA, USA), with 300-bp paired-end reads and 30-fold genome coverage. The genome sequencing generated 450,167 reads. The initial quality of the raw sequencing data was checked using FastQC (http://www.bioinformatics.babraham.ac.uk/projects/fastqc). The raw reads and adapter contaminations were trimmed with Sickle version 0.991 (https://github.com/najoshi/sickle) and Scythe version 1.33 (https://github.com/vsbuffalo/scythe), respectively. Assembly was performed using SPAdes version 3.13 (6) using k-mer sizes set to 21, 33, 55, 77, 99, and 127 and the careful pipeline option. Default parameters were used for all software unless otherwise specified. Structural gene prediction and functional annotation were performed using the Rapid Annotations using Subsystems Technology (RAST) server (7). Gene sequences were then compared using the online BLAST server (8). The total size of the draft assembly was 5,274,200 bp, distributed into 91 contigs with an *N_50_* value of 188 kb. The genome has an average GC content of 35.2% and contains 5,552 putative coding sequences and 112 putative RNA genes.

RAST annotation of the Bacillus cereus TN10 genome revealed the presence of chromate transporters, which are thought to be involved in Cr^6+^ resistance (9). A number of genes involved in sulfur assimilation and transport and in iron sequestering were also detected. The TN10 genome harbors genes encoding different reductases, including dihydrolipoamide dehydrogenase. Other genes and gene clusters involved in arsenic (ACR3), copper (kBcr/CflA family transporter), and cobalt-zinc-cadmium resistance were also detected in the TN10 draft genome.

### Data availability.

This whole-genome shotgun project has been deposited in DDBJ/ENA/GenBank under accession no. QFWR00000000.1. The raw reads have been deposited in the NCBI SRA database under accession no. SRX7683794 and SRX7683795.
